# Macrophage-induced immunomodulation in oral tissue repair and regeneration: Recent advances and future perspectives

**DOI:** 10.1016/j.jare.2025.11.057

**Published:** 2025-11-29

**Authors:** Yujie Wang, Jing Mao, Yifan Wang, Rui Wang, Dajie Duan, Zhenyuan Liu, Xiaohan Hu, Zili Yu, Xin Shi

**Affiliations:** a Center of Stomatology, Tongji Hospital, Tongji Medical College, Huazhong University of Science and Technology, Wuhan 430030, China; b School of Stomatology, Tongji Medical College, Huazhong University of Science and Technology, Wuhan 430030, China; c Hubei Province Key Laboratory of Oral and Maxillofacial Development and Regeneration, Wuhan 430022, China; d State Key Laboratory of Oral and Maxillofacial Reconstruction and Regeneration, Key Laboratory of Oral Biomedicine Ministry of Education, Hubei Key Laboratory of Stomatology, School and Hospital of Stomatology, Wuhan University, Wuhan 430079, China; e Department of Oral and Maxillofacial Surgery, School and Hospital of Stomatology, Wuhan University, Wuhan 430079, China; f Outpatient Department Office, Tongji Hospital, Tongji Medical College, Huazhong University of Science and Technology, Wuhan 430030, China

**Keywords:** Macrophages, Exosomes, Periodontitis, Pulpitis, Peri-implantitis, Jawbone defects

## Abstract

**Objectives:**

Maintaining immune balance plays crucial roles in the development and prognosis of injured oral tissues. Serving as critical components of immune system, macrophages are essential in accelerating oral tissue repair and regeneration. This multipart review intends to present a comprehensive overview of recent advances and future perspectives of macrophage plasticity and polarization in oral tissue repair and regeneration, thereby enhancing the understanding of macrophage-induced immunotherapeutic effects and underlying mechanisms in oral diseases.

**Methods:**

The primary electronic databases consulted for this review included PubMed, Web of Science, and Scopus. The literature search strategy employed a comprehensive combination of keywords such as “macrophage polarization”, “M1”, “M2”, “oral tissue repair”, “periodontitis”, “pulpitis”, “osseointegration”, “jawbone defects”, and “immunotherapy”. Both original research articles and authoritative review papers were selected. Furthermore, the reference lists of retrieved articles were manually examined to identify additional pertinent publications.

**Results:**

Macrophages are derived initially from hematopoietic stem cells in the bone marrow and embryonic yolk sac progenitor cells. They encompass several polarization phenotypes, including M1, M2a, M2b, M2c, and M2d, and exert multifaceted functions by secreting soluble factors and insoluble exosomes. Encouragingly, robust immunotherapeutic potential and distinctive regulatory mechanisms of macrophages have been revealed for a variety of oral diseases, providing a breadth of perspectives and insights into the repair and regeneration of oral tissues.

**Conclusions:**

Orderly and sequential polarization of macrophages has been proposed as novel immunotherapeutic strategies and promising directions for treating oral diseases. An in-depth exploration of hidden mechanisms may contribute to structural regeneration and functional reconstruction of oral tissues.

## Highlights

• This review provides a comprehensive analysis of the ontogeny and tissue-specific distribution of macrophages.
• The diverse polarization phenotypes of macrophages and their respective secretory profiles are discussed in detail.
• Particular emphasis is placed on macrophage regulatory mechanisms and immunotherapeutic potential in various oral pathologies.
• Perspectives on modulating macrophage polarization are highlighted to propose novel immunotherapies for managing oral diseases.

## Graphical abstract

**Figure:**
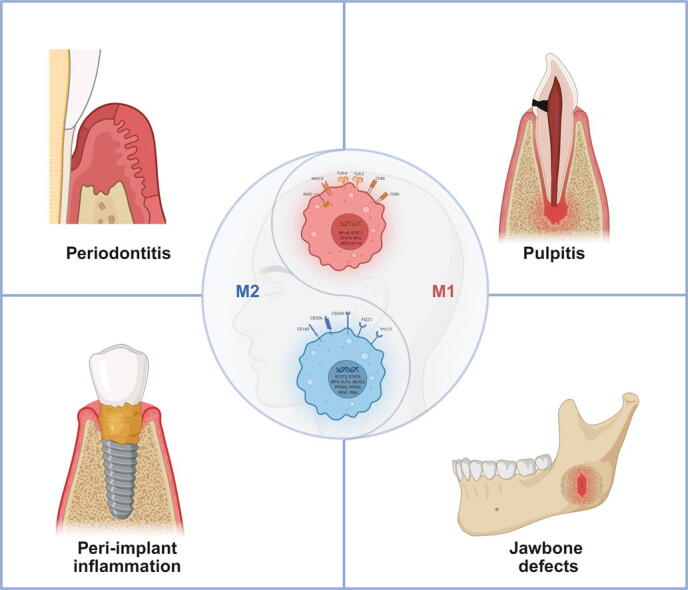

## Introduction

The oral cavity harbors diverse pathogenic and opportunistic microbial communities. Oral diseases encompass a broad spectrum of pathological conditions that affect both soft and hard tissues within the oral and maxillofacial regions, including periodontal disease, pulpitis, jaw injuries, peri-implantitis, oral mucosal diseases, and cleft lip and/or palate [1]. These diseases pose significant global public health concerns due to their high prevalence and the risk of progression into chronic infections if not appropriately managed [2]. Furthermore, accumulating evidence supports bidirectional associations between certain oral and systemic diseases. For instance, individuals with type 1 diabetes frequently exhibit elevated colonization levels of specific pathogenic microorganisms in their oral microbiota, such as Porphyromonas gingivalis, Prevotella intermedia, and Actinomyces species, which heighten their susceptibility to periodontal diseases [3]. Conversely, periodontal inflammation can adversely influence blood sugar control. In consequence, there is an urgent need to identify potential diagnostic and therapeutic targets for oral diseases.

Macrophages are ubiquitously distributed throughout the body, residing in diverse anatomical locations, including the brain, liver, bone marrow, alveoli, and lymph nodes. Their precursors, monocytes, circulate in the bloodstream and occupy the bone marrow prior to differentiating into macrophages upon infiltrating various tissues. Macrophages execute multifaceted functions ranging from pathogen clearance and antigen presentation to orchestration of tissue repair and regeneration and the maintenance of microenvironmental homeostasis [4]. Numerous inflammatory diseases, such as pneumonia, intestinal inflammation, hepatitis, wound healing-associated inflammation, and periodontitis, have been closely associated with macrophage activity [5]. Macrophages are traditionally classified into M1 and M2 phenotypes, reflecting their distinct polarization states and functional profiles. However, it is crucial to recognize that macrophage phenotypes present in vivo are considerably more complex and often display mixed characteristics with monocytes. In functional terms, M1 macrophages are distinguished by their pro-inflammatory properties, while M2 macrophages exhibit anti-inflammatory activities. Recent advancements have led to a more refined categorization, with M2-like macrophages discernible as subtypes, namely, M2a, M2b, M2c, and M2d, contingent upon the nature of the stimuli [6]. Pro-inflammatory mediators such as lipopolysaccharide (LPS) and interferon-γ (IFN-γ) induce the polarization of M1 macrophages, eliciting the secretion of various pro-inflammatory cytokines and chemokines, including tumor necrosis factor-α (TNF-α), interleukin-6 (IL-6), IL-1α, IL-1β, and C-X-C motif chemokine ligand 9 (CXCL9) [7]. Conversely, M2 macrophages are activated by anti-inflammatory mediators such as IL-4 and IL-13, which stimulate the release of anti-inflammatory factors, including transforming growth factor-β (TGF-β), IL-10, C–C motif chemokine ligand 1 (CCL1), CCL17, and CCL18, participating in anti-inflammatory responses [8]. Functionally, classically activated M1 macrophages are primarily engaged in phagocytosis, MHC II antigen presentation, and the generation of reactive oxygen species [9]. In contrast, M2 macrophages promote extracellular matrix production, cell proliferation, and angiogenesis, thereby facilitating tissue remodeling and repair [10]. Consequently, the diverse phenotypes and secretory profiles of macrophages hold significant promise in the realm of oral disease prevention and treatment.

Nonetheless, the precise mechanisms underlying macrophage polarization in oral pathologies, the intricate regulatory networks governing phenotypic plasticity, and the dynamic shifts in macrophage states throughout disease progression remain insufficiently elucidated. Within the oral microenvironment, a diverse array of cytokines, exosomes, resident microbiota, and their metabolic products converge to modulate macrophage phenotype and function. Accordingly, illustrating the polarization patterns and hidden molecular signaling pathways of macrophages in oral diseases is critical to deepen our understanding of immunopathogenesis and to establish a scientific rationale for the development of macrophage-targeted immunotherapeutic strategies. The present review aims to provide a comprehensive and systematic analysis of macrophage phenotypic profiles, regulatory pathways, and emerging interventional approaches in prevalent oral disorders, thereby advancing the modulation of the oral immune microenvironment.

## Origin and distribution of macrophages

In 1908, Nobel Prize laureate Ilya Metchnikov made a seminal discovery in the field of immunology by identifying phagocytic cells in both unicellular organisms and vertebrates, which were imperative in recognizing, engulfing, and degrading cellular debris and pathogens [1,11,12]. He subsequently designated these cells as macrophages. Initially, the scientific community held a limited understanding of the origin of macrophages, with the prevailing belief that they were exclusively reliant on the continuous replenishment of monocytes. However, advancing studies have elucidated two principal sources of macrophage populations: first, monocytes, which are derived from hematopoietic stem cells in the bone marrow and then migrate into peripheral tissues; second, embryonic yolk sac progenitor cells that can differentiate into self-renewing, tissue-resident macrophages independently of monocytes throughout adulthood [13,14]. As research evolves, an increasing body of evidence underscores that macrophages possess critical impacts on orchestrating pathogen defense, facilitating intercellular immune communication, supporting tissue development, and maintaining homeostatic balance within the organism.

However, as ubiquitous immune cells, macrophages demonstrate distinct distributions and functional mechanisms across diverse physiological niches, including lymph nodes, alveolar walls, bone marrow, liver, and spleen. These cellular populations display intricate heterogeneity, differentiating into subpopulations with specialized roles, such as microglia, osteoclasts, alveolar macrophages, splenic histiocytes, and Kupffer cells [13]. For instance, the critical effects of macrophages on mammary gland development and pancreatic branching morphogenesis are particularly noteworthy [15]. In the cardiovascular system, macrophages serve multiple functions, including the construction of vascular walls [16] and the stabilization of cardiac rhythms [17], both of which are essential for maintaining cardiovascular health. In this context, recent evidence has revealed that the flavonoid isoorientin alleviated atherosclerosis by suppressing macrophage pyroptosis via the KDM4A-SKP1-NLRP3 axis, highlighting a novel paradigm for macrophage-targeted therapeutic intervention in inflammatory diseases [18]. Furthermore, macrophages are indispensable during organ-specific pathologies, contributing to the maintenance of tissue homeostasis and promoting tissue repair and regeneration. Within the hepatic environment, Kupffer cells not only exhibit self-renew capabilities but also perform pivotal biological mechanisms in the pathogenesis of acute and chronic liver failure, fibrosis, viral hepatitis, and hepatocellular carcinoma [19]. Additionally, microglia in the central nervous system are instrumental in regulating myelin clearance, debris removal, and cytokine release, all of which are crucial for the repair and recovery of neurons following injury [20]. In light of these findings, macrophages exhibit remarkable diversity and complexity in their functions across different organs and pathophysiological conditions. The multifaceted roles of macrophages underscore their significance in regulating tissue homeostasis and participating in organogenesis and emphasize the imperative for continued research to better elucidate their implications for therapeutic interventions in various disease contexts.

## Polarization, classification, and secretory role of macrophages

**Fig. 1:**
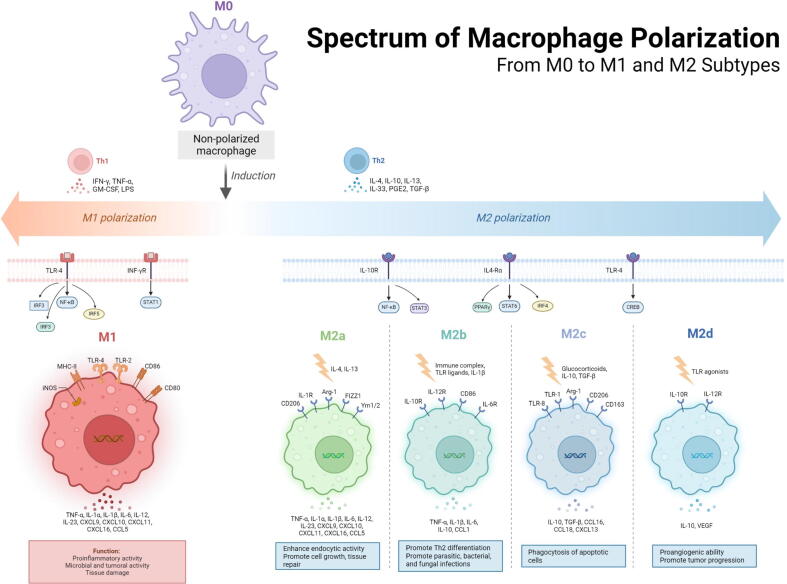
Macrophages undergo polarization in response to diverse stimulating factors and exhibit distinctive functions in pathophysiological environments. Upon exposure to Th1 cytokines (e.g., IFN-γ, TNF-α) or microbial-derived products (e.g., LPS), M0 macrophages differentiate towards the classically activated M1 phenotypes. This state is characterized by the elevated expression of surface markers, including CD40, CD64, CD68, CD80, CD86, and HLA-DR, as well as robust secretion of pro-inflammatory cytokines such as TNF-α, IL-1β, IL-6, IL-12, and IL-23. M1 macrophages are indispensable for pathogen clearance and antitumor immunity, but their dysregulated or sustained activation can precipitate collateral tissue damage. Conversely, stimulation with Th2 cytokines (e.g., IL-4, IL-13) or other anti-inflammatory signals induces transformation of M0 macrophages into the alternatively activated M2 phenotype, which is primarily implicated in inflammation resolution, tissue remodeling, and immunoregulatory processes. Due to their functional heterogeneity, M2 macrophages are further subclassified into distinct subsets according to their specific inducing stimuli, phenotypic markers, and biological functions: M2a (Wound healing): Elicited by IL-4 and IL-13; characterized by expression of CD163, CD206, and ARG1; secrete TGF-β, CCL17, and CCL18; primarily involved in tissue repair and fibrogenesis. M2b (Regulatory): Induced by immune complexes and TLR agonists; express CD86 and CD14; produce high levels of IL-10; mediate immunoregulatory responses. M2c (Acquired deactivation): Stimulated by IL-10, TGF-β, and glucocorticoids; share CD163 and CD206 expression with M2a; secrete IL-10 and CCL16; participate in the suppression and clearance of apoptotic cells. M2d (Tumor-associated): Induced by adenosine and IL-6; distinguished by elevated VEGF production and secretion of IL-10 and TGF-β; promote angiogenesis and tumor progression.

Mature macrophages exhibit substantial plasticity and heterogeneity in their phenotype and function. This enables them to adapt to and respond to diverse environmental signals. Macrophages are divided into two main activation states, M1 and M2, based on Th1 and Th2 helper T cell polarization concepts (Fig. 1). Mills et al. initially proposed this classification, which has since been broadly used to describe the predominant macrophage subsets [21]. Macrophages can activate different signaling pathways upon sensing changes in the local tissue microenvironment, polarizing into specific phenotypes and transitioning between phenotypes under certain conditions. The ratio of M1 and M2 macrophages closely correlates with numerous physiological and pathological states [[22], [23], [24]].

M1 macrophages, also known as classically activated pro-inflammatory macrophages, are polarized predominantly in response to granulocyte–macrophage colony-stimulating factor (GM-CSF), toll-like receptor (TLR) ligands, including LPS, and Th1 cytokines such as IFN-γ [23,25]. These cells are characterized by robust expression of pro-inflammatory cytokines, including CD40, CD64, CD68, CD80, CD86, and human leukocyte antigen-DR [26]. Moreover, M1 macrophages possess pronounced antigen-presenting capabilities, thereby playing critical roles in the amplification of inflammatory responses, the eradication of pathogenic microorganisms, and the mediation of antitumor immunity [27]. Upon activation, M1 macrophages can release large amounts of pro-inflammatory cytokines, including TNF-α, IL-1β, IL-6, IL-12, and IL-23 [28]. Notably, the initial signals that contribute to this inflammatory milieu can be attributed to tissue-resident cells. For instance, research on the novel allergen Der p 22 has delineated its capacity to induce apoptosis in pulmonary epithelial cells while concurrently stimulating the secretion of pro-inflammatory cytokines, such as IL-6, IL-8, and GM-CSF. These epithelial cell-derived mediators are pivotal in orchestrating the recruitment and activation of immune cells, including M1 macrophages, at inflammatory sites, thereby intensifying the inflammatory cascade [29]. Under normal physiological conditions, the proportion of M1 macrophages is precisely controlled to preserve immune homeostasis. However, during inflammatory responses, there is a marked expansion of M1 macrophage population [24]. In the early stage of inflammation, M1 cells facilitate the clearance of invading pathogens, bacteria, and cellular debris via phagocytosis. Nevertheless, dysregulated or excessive secretion of pro-inflammatory cytokines can trigger heightened inflammatory responses, culminating in tissue damage and impaired wound healing [26,30].

M2 macrophages, representing alternatively activated anti-inflammatory macrophages, play key roles in inflammation resolution and tissue repair and regeneration. Furthermore, they are involved in various chronic infection processes. M2 macrophages can be activated by IL-4, IL-13, IL-17, immune complexes, mesenchymal stem cells (MSCs), glucocorticoids, and macrophage colony-stimulating factors [31]. Their primary function is to secrete anti-inflammatory cytokines, aiding in regulating the inflammatory response. These cells express high levels of CD163, CD206, CD209, scavenger receptor A (SR-A), SR-B1, chemokine CC receptor 2, CXC chemokine receptor 1 (CXCR1), and CXCR2 [13]. Depending on the microenvironment and stimuli, M2 macrophages are divided into four subtypes: M2a, M2b, M2c, and M2d [32]. M2a macrophages, derived from unpolarized macrophages under IL-4 and IL-13 stimulation, are associated with allergic reactions. They express high levels of CD163, CD206, IL-1R, Fizz1, arginase-1, and Ym1/2 and secrete factors needed for tissue repair, including transforming growth factor-β (TGF-β), insulin-like growth factor, fibronectin, and chemokines such as CCL13, CCL17, CCL18, CCL22, and CCL24. M2a macrophages recruit eosinophils, basophils, and Th2 cells to participate in the immune response [22,26,33]. M2b macrophages, induced by immune complexes, TLRs, and IL-1R, participate in anti-inflammatory responses and immune regulation. They express high levels of CD14 and CD80 and secrete anti-inflammatory cytokines, including IL-10. M2b macrophages can recruit regulatory T cells, thus aiding in countering excessive inflammatory responses [34]. M2c macrophages, induced by IL-10, TGF-β, and glucocorticoids, are critical in inhibiting immune responses and promoting tissue repair. These cells express high levels of CD163, CD206, and advanced glycation end-product receptors and secrete significant quantities of IL-10, TGF-β, CCL16, and CCL18. They can recruit eosinophils and resting T cells, regulating related biological processes through complex cellular interactions [35,36]. M2d macrophages, or tumor-associated macrophages, are induced by adenosine A2 receptors, leukemia inhibitory factors, and IL-6. They suppress inflammatory responses, promote angiogenesis, and facilitate cancer cell progression and invasion [37]. Additionally, they express abundant levels of CD163 and vascular endothelial growth factor (VEGF) and secrete large amounts of IL-10, TGF-β, CXCL10, CXCL16, and CCL5 [26].

**Fig. 2:**
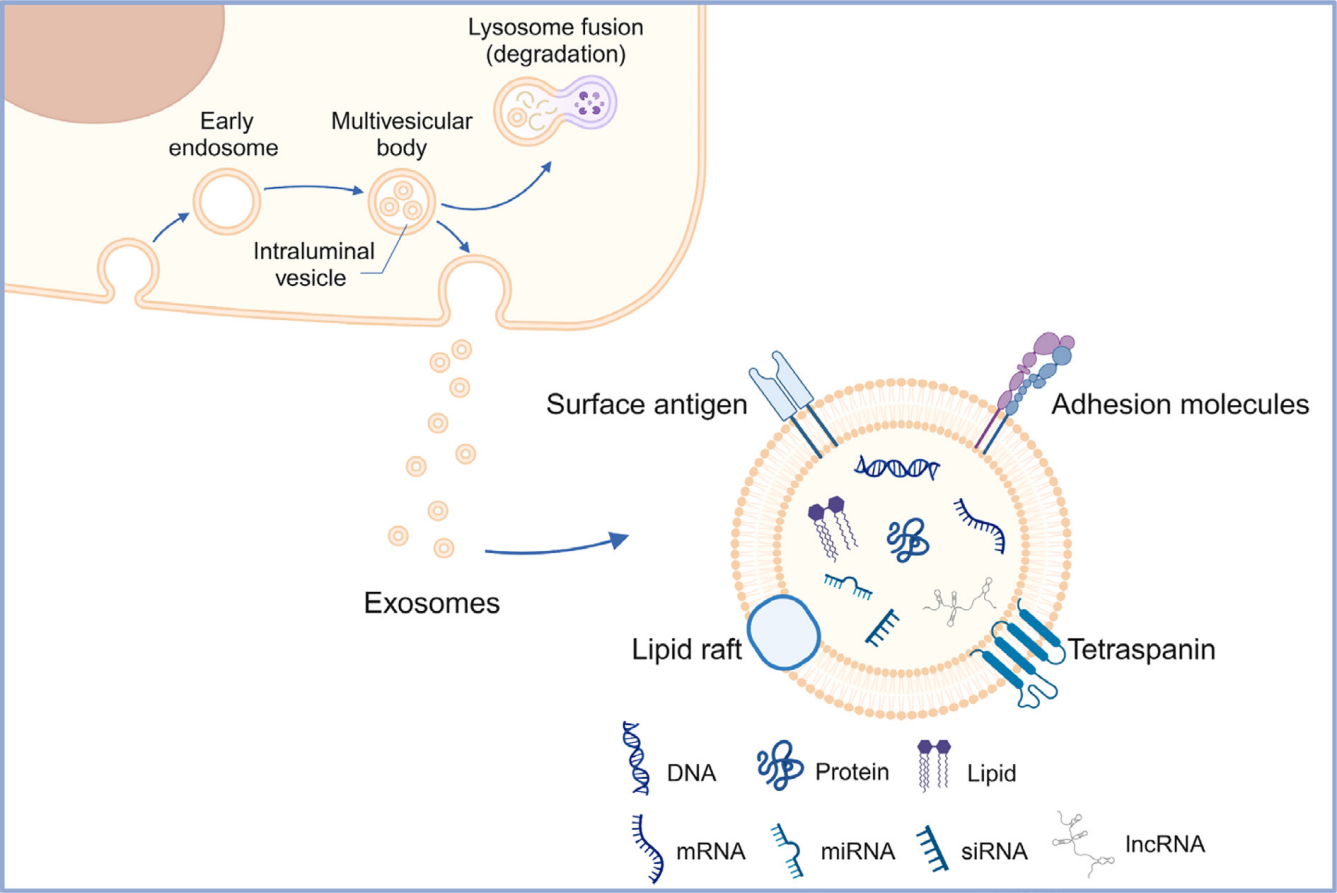
Schematic illustration of exosome biogenesis and structure composition. Exosomes are lipid-bilayer extracellular vesicles derived from endosomes with an average diameter of 30–150 nm. They are released into the extracellular milieu through the fusion of the multivesicular body with the cytoplasmic membrane. They encapsulate various biomolecules, such as nucleic acids, proteins, and lipids, which play a crucial role in mediating intercellular communication in both physiological and pathological contexts.

Of particular significance, in addition to the aforementioned soluble factors, macrophages, emerging as prototypical secretory cells, also generate extracellular vesicles (EVs), which serve pivotal roles in immunomodulation [38]. EVs can originate from a wide range of cell types and are detectable in various biological fluids such as plasma, serum, saliva, urine, breast milk, cerebrospinal fluid, amniotic fluid, and pleural effusions [[39], [40], [41]]. They are categorized into three major types based on their size, biogenesis pathways, and functional attributes, including apoptotic bodies (500–2000 nm), microvesicles (200–1000 nm), and exosomes (30–150 nm) [42]. Notably, exosomes, representing the smallest nanoscale EVs, are enriched in specific cell proteins, lipids, and various types of RNAs, encompassing mRNAs, miRNAs, and other non-coding RNAs. These nanovesicles mediate the transport of molecular cargo and facilitate intercellular communication, thereby exerting profound influences on a series of physiological and pathological processes, as well as essential biological behaviors [43]. The biogenesis, cargo packaging, and secretion of exosomes are meticulously orchestrated processes predominantly governed by the endocytic pathway. This pathway commences with the invagination of the plasma membrane to form early endosomes, which internalize membrane proteins and extracellular components [8] (Fig. 2). Then, early endosomes progress through maturation stages to become late endosomes, which transform into multivesicular bodies (MVBs) containing intraluminal vesicles (ILVs). In consequence, these MVBs can either merge with lysosomes or autophagosomes for degradation or fuse with the plasma membrane to release ILVs as exosomes through cytoskeletal and microtubule networks. To be specific, exosomes derived from different macrophage phenotypes convey versatile biological signals and perform unique functions. Current research primarily focuses on three categories of macrophage-derived exosomes, namely, non-polarized M0-derived exosomes (M0-Exos), classically activated M1-derived exosomes (M1-Exos), and polarized M2-derived exosomes (M2-Exos) [44]. As revealed by Liu et al., M1 macrophages released a significant amount of pro-inflammatory M1-Exos following myocardial infarction, which encapsulated a substantially elevated level of miRNA-155 [45]. Mechanistically, miRNA-155 could be internalized by endothelial cells, which subsequently suppressed Sirt1/AMPKα2-eNOS and RAC1-PAK2 signaling pathways, ultimately leading to the inhibition of endothelial angiogenesis, aggravation of myocardial injury, and impediment of cardiac healing. In the context of diabetic femoral fracture, Wang et al. illustrated for the first time that the enhanced infiltration of M1 macrophages contributed to the impaired cartilage formation and mineralization, thereby hindering fracture healing [46]. Encouragingly, the local administration of M2-Exos has been shown to facilitate the phenotypic conversion of macrophages from M1 into M2 via the PI3K/AKT pathway, which ultimately expedited diabetic fracture healing. In line with this, additional investigations have underlined the therapeutic promise of M2-Exo in a viral myocarditis model, where they attenuated tissue injury by promoting the repolarization of M1 to M2 macrophages [47]. This process was mediated by lncRNA AK083884, encapsulated within M2-Exo, which orchestrated metabolic reprogramming through the PKM2-HIF-1α axis and activated the SOCS2/STAT6 signaling pathway to drive phenotypic switching. Hence, exosomes derived from macrophages possess critical effects on immune surveillance, signal transduction, and disease progression, shedding bright light on their considerable potential in advancing disease diagnostics, therapeutic interventions, and clinical translation.

Efficient isolation and purification of exosomes serve as crucial prerequisites for investigating their biological functions. Current methodologies primarily encompass techniques based on physical properties, chemical precipitation, and affinity-based capture [48]. Among these, ultracentrifugation, widely considered as the “gold standard” for exosome isolation, employs differential centrifugation to enrich exosomes from cell culture supernatants or various bodily fluids. Although this method offers broad applicability and is relatively cost-effective, it is characterized by high shear forces that may compromise the integrity of exosome membranes and co-isolate other vesicular components such as apoptotic bodies [49]. To enhance purity of isolated exosomes, density gradient centrifugation with sucrose or iodixanol media has been exploited to facilitate refined purification through differential vesicular buoyancy, providing particularly advantageous for complex biological specimens such as serum or saliva [50]. For research requiring small sample volumes or the preservation of bioactivity, size exclusion chromatography achieves effective separation of exosomes within the range of 30–150 nm via molecular sieving while maintaining their native structural and functional integrity [51]. In recent years, polymer precipitation methods, especially those utilizing polyethylene glycol reagent kits, have gained popularity due to their operational simplicity and equipment-free protocols. However, these methods require careful monitoring to prevent co-precipitation of protein aggregates or lipoproteins, underscoring the importance of validation through Western Blot (WB) or nanoparticle tracking analysis (NTA) using specific markers [52]. Immunoaffinity capture enables precise enrichment of exosomal subpopulations by targeting surface markers, such as CD63 or CD81 antibodies and M2 macrophage-specific CD206, albeit potentially missing heterogeneous populations due to epitope masking [50]. Once isolated, exosomes must be validated for their physical characteristics, molecular markers, and cargo composition through multimodal techniques. Morphologically, transmission electron microscopy provides ultrastructural visualization of characteristic bilayered membranes, while scanning electron microscopy reveals detailed surface topography [53]. Particle size and concentration analysis are typically performed using NTA or dynamic light scattering to confirm that the vesicles in the sample fall within the desired size range and to exclude the contamination of apoptotic bodies [54]. Of importance, molecular marker validation necessitates a combination of WB detection of exosome-specific proteins (e.g., TSG101, CD9, and CD63) alongside specific source markers (e.g., CD206 and Arg1). Notably, flow cytometry can be utilized to quantitatively analyze the surface markers of exosomes by conjugating CD63 antibodies to magnetic beads [55]. Furthermore, high-throughput cargo analysis techniques, including proteomics (e.g., LC-MS/MS) to identify immunomodulatory factors such as TGF-β) [56], as well as RNA sequencing for assessing miRNA function [57], further elucidate the bioactive molecular mechanisms of exosomes. Moreover, lipidomics and metabolomics can provide additional insights into their complex biological roles [58].

In summary, M1, M2, and their subtypes are essential in regulating inflammation, tumor progression, and tissue repair, providing critical insights for understanding disease mechanisms and developing therapeutic strategies.

## Regulatory mechanism and therapeutic potential of macrophages in oral diseases

### Periodontitis

Periodontitis, a chronic infectious inflammatory disorder, is intricately associated with microecological dysbiosis and is precipitated primarily by persistent destruction of periodontal supporting tissues in response to plaque biofilm accumulation. It is distinguished by a host-mediated inflammatory response to microbial insults, which leads to the progressive loss of periodontal attachment. Clinically, periodontitis manifests as irreversible destruction of tooth-supporting tissue and the formation of periodontal pockets [59]. The underlying inflammation arises from complex interactions between subgingival biofilm-associated pathogenic microorganisms and various immune cell populations within oral tissues, which constitute a principal driver of macrophage polarization. Specifically, biofilm-derived pathogens and their molecular components, such as LPS, activate pattern recognition receptors on macrophages, which, in concert with cytokines such as IFN-γ secreted by Th1 cells, promote pro-inflammatory M1 polarization that predominates during tissue destructive phases. In contrast, alterations in the microbial biofilm and the tissue microenvironment during resolution phases, alongside the presence of anti-inflammatory cytokines such as IL-4 and IL-13, drive a shift towards M2 polarization, thereby supporting tissue repair and the resolution of inflammation [60]. Polymorphonuclear cells penetrate the highly permeable epithelial barrier of the oral mucosa to engage in immune responses. Additionally, macrophages, T cells, B cells, innate lymphoid cells, and dendritic cells are integral to orchestrating the immune defense network within periodontal tissues [61,62]. Of great significance, the host defense response exerts dual, and sometimes paradoxical, effects in the context of periodontal disease progression. While macrophages potentiate antibacterial defenses and release cytokines to coordinate multicellular immune responses against invading pathogens [63], the heterogeneity and diversity of cytokines produced can also exacerbate tissue destruction, thereby amplifying periodontal pathology [64]. A nuanced understanding of the distinct roles of macrophage polarization phenotypes in the initiation and progression of periodontitis is therefore essential for elucidating disease pathogenesis and informing the development of targeted therapeutic strategies.

#### The role of M1 macrophages in tissue destruction

In periodontal inflammation, M1 macrophage polarization is critical, primarily triggered by Th1 cell-secreted IFN-γ and microbial-associated factors such as LPS [65,66]. Periodontal pathogens activate macrophage surface receptors, including CD14, TLRs, and nucleotide-binding and oligomerization domain-like receptors, inducing M1 differentiation and contributing to inflammation regulation [67]. Cytokines secreted by M1, such as prostaglandin E2 (PGE2), matrix metalloproteinase 1 (MMP-1), MMP-2, IL-1β, TNF-α, IL-6, IL-12, cyclooxygenase, and inducible nitric oxide synthase, are essential in periodontitis progression and bone resorption. As revealed by Ruiz-Heiland et al., PGE2 was capable of reducing osteoblast survival and increasing osteoclast formation, consequently causing alveolar and cementum destruction [68]. Lew et al. illustrated that IL-1β and IL-6 released by M1 prompted human gingival fibroblasts (HGFs) to express MMP-1 under hyperglycemia, leading to collagen fiber damage and worsening periodontitis [69]. Besides, IL-1β could augment the IL-6 response of HGFs, showing a synergistic role in periodontal progression [70]. IL-6 activated MMPs to degrade the extracellular matrix, causing alveolar bone erosion [71]. Notably, the application of IL-6 receptor inhibitors showed good clinical potential for preventing bone resorption and reducing periodontal inflammation [72]. From a clinical perspective, IL-6 receptor inhibitor treatment in patients with periodontitis considerably improved gingival health, as evidenced by the remarkable reduction in gingival index, decreased bleeding on probing, and diminished probing depth, all key indicators of periodontal improvement [73]. According to Checchi et al., MMP-2 could be directly synthesized and secreted by M1 macrophages, leading to the destruction and degradation of periodontal connective tissue, which played a critical role in periodontal tissue degradation [74]. Additionally, M1 macrophages were able to enhance tissue destruction by activating Th17 cells. A close relationship exists between Th17 and M1 cells, exhibiting robust proliferation and differentiation potential in chronic periodontal inflammation. Th17 cells predominantly function by promoting inflammation and recruiting neutrophils, characterized by their production of the vital cytokine IL-17 [75]. A previous study reported that elevated levels of local Th17 cells and IL-17 correlated positively with the severity and clinical parameters of periodontal destruction [76]. During periodontitis progression, activation of the NLRP3 inflammasome signaling pathway promoted M1 polarization, leading to the secretion of inflammatory cytokines such as TNF and IL-1β, thereby accelerating periodontitis advancement [77]. Collectively, these findings suggest that M1 macrophages are essential in periodontal inflammation by secreting various cytokines and activating related signaling pathways, inducing periodontal tissue destruction. However, further research is warranted to explore more detailed molecular mechanisms of M1 hidden in periodontitis.

#### The role of M2 macrophages in inflammation resolution and tissue repair

On the contrary, M2 macrophages primarily mitigate inflammation during periodontitis progression and promote tissue repair by producing IL-10 and reducing IL-6 expression [78]. M2 macrophages possess notable anti-inflammatory capability due to their suppression of NF-κB and other inflammatory pathways [79]. As inflammation advances, M2 cells counteract M1 cells by secreting various reparative mediators such as IL-4, IL-10, IL-13, and TGF-β, thus regulating the anti-inflammatory response and facilitating wound healing and tissue regeneration while maintaining local homeostasis [[80], [81], [82]]. M2 polarization is largely driven by IL-4 and IL-13 [64]. Moreover, IL-10 is also essential for M2 polarization and function regulation. On one hand, IL-10 can convert immature monocytes into M2 macrophages [83] and enable M2 to express high levels of IL-10 [84]. On the other hand, IL-10 holds great potential to enhance scavenger receptor-A expression while inhibiting the expression of CD14, TLR4, and major histocompatibility complex II [85]. In addition to IL-10, other cytokines secreted by M2 cells demonstrate anti-inflammatory effects via diverse mechanisms. For instance, the IL-1 receptor antagonist produced by M2 has been demonstrated to suppress IL-1 and TNF-α-induced osteoclast activity [84]. Furthermore, M2 can secrete immune-regulating molecules such as CCL1 and CCL16 to maintain immune balance and homeostasis [86]. As documented by Zhuang et al. in mouse models of periodontitis induced by *Porphyromonas gingivalis* and ligatures, the employment of CCL2 effectively promoted M2 polarization and diminished the M1 to M2 ratio in periodontal tissue, consequently reducing osteoclast numbers and decreasing alveolar bone resorption [87]. Bone marrow MSCs (BMSCs) cultured in the conditioned medium derived from M2 macrophages (M2-CM) exhibit markedly upregulated expression of osteogenesis-related genes, including *runt-related transcription factor 2 (RUNX2), collagen type I (COL1), alkaline phosphatase (ALP),* and *osteocalcin (OCN)* [88]. Meanwhile, their proliferation and mineralization capacities were augmented substantially. Conversely, macrophages cultivated under M2-CM conditions displayed remarkably diminished expression of osteoclastic-related genes, such as *NFATc1, JDP2, TRAP,* and *CTSK*, accompanied by repressed TRAP staining. These findings indicated that M2-CM provided additional advantages in inhibiting osteoclast formation via paracrine mechanisms. In this regard, concentrating on secretory exosomes, Chen et al. demonstrated that M2-Exos exerted a dose-dependent modulation on the expression of osteogenic and osteoclastic-related genes, thereby promoting osteogenic differentiation while inhibiting osteoclast differentiation [89]. In a murine model of periodontitis, M2-Exos were uncovered to facilitate the upregulation of IL-10 expression in BMSCs and bone marrow-derived macrophages through the direct transfer of exosomal *IL-10* mRNA, thus activating the IL-10/IL-10R pathway and enhancing alveolar bone regeneration. Concurrently, recent research signified that M2-Exos elicited a positive impact on the osteogenic differentiation of human periodontal ligament stem cells (hPDLSCs) in the presence of LPS-induced inflammation, which was evidenced by the elevated expression of ALP, COL1, and RUNX2 in mRNA and protein levels [90]. Intriguingly, M2-Exos have been corroborated in vitro to restrain the level of receptor activator of nuclear factor κB ligand (RANKL), a decisive regulator of osteoclast activity. Consistent with these findings, in a rat model of ligature-induced periodontitis, micro-CT analysis further ascertained that M2-Exos were capable of attenuating periodontal bone loss. From a histological perspective, HE staining and Masson’s trichrome staining unveiled an increased thickness of epithelial cell layers, reduced inflammatory cell penetration, and improved organization and density of elastic and collagen fibers, as well as enhanced alveolar bone formation. Additionally, TRAP staining validated a notable decrease in osteoclast infiltration. Besides, immunohistochemistry analysis revealed a significant decrease in the expression of endoplasmic reticulum stress and apoptosis-related proteins, including glucose-regulated protein 78, C/EBP-homologous protein, and caspase-3, in periodontal tissues following M2-exo treatment. Collectively, M2-Exos have shown promise in mitigating endoplasmic reticulum stress-induced apoptosis and restoring impaired hPDLSC functions in inflammatory conditions, paving the way for periodontal regeneration. Hence, M2 macrophages are essential in promoting bone integration and inhibiting bone resorption during periodontitis. Regulating the ratio of M1 to M2 macrophages contributes to maintaining the balance between immunodefense and inflammatory responses, providing a novel therapeutic target for periodontitis treatment.

#### Key signaling pathways governing macrophage polarization in periodontitis

**Fig. 3:**
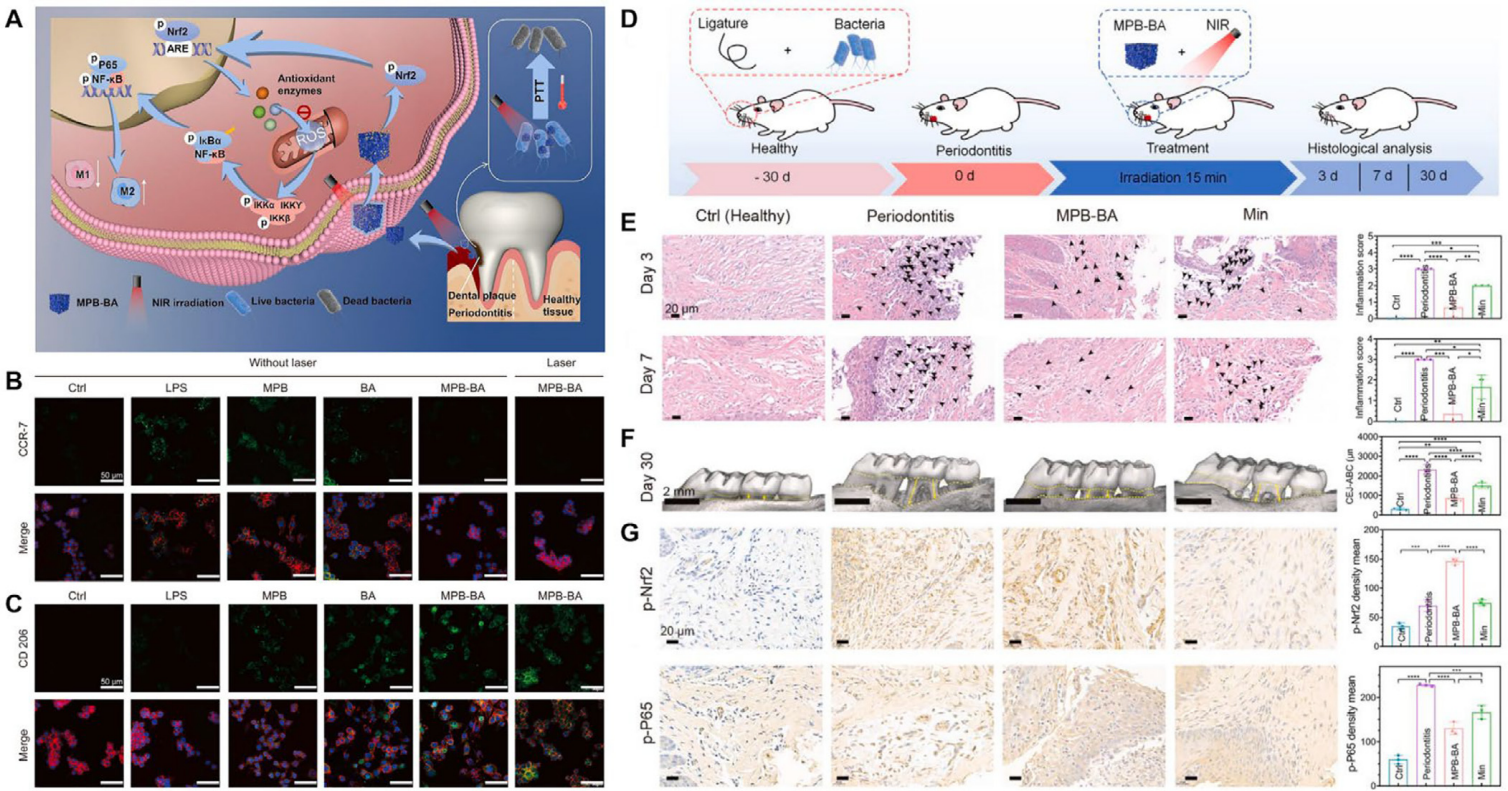
MPB-BA alleviated rat periodontitis by promoting macrophage transition from M1 to M2 phenotype. (A) Schematic representation detailing the mechanisms underlying the immunomodulatory property of MPB-BA in the management of periodontitis. (B) As revealed by immunofluorescence staining, the fluorescent intensity of M1 marker CCR-7 in macrophages was substantially attenuated when subjected to MPB-BA treatment with or without laser irradiation. (C) In sharp contrast, the fluorescent signal of M2 marker CD206 was remarkably enhanced. (D) Schematic illustration of MPB-BA treatment process in rats with periodontitis. The group treated by minocycline hydrochloride (Min), a clinically commonly employed antibiotic, was designed as a positive control. (E) HE staining confirmed that MPB-BA considerably suppressed the infiltration of inflammatory cells after 3 and 7 days of treatment, which was superior to the Min group. (F) Micro-CT examination confirmed that MPB-BA elicited a marked inhibitory role in mitigating alveolar bone loss. (G) Mechanistically, according to immunohistochemical staining for p-Nrf2 and p-P65, MPB-BA possessed outstanding anti-inflammatory effect by elevating p-Nrf2 protein level while inhibiting p-P65 level, indicative of the importance of Nrf2/NF-κB signaling pathway in MPB-BA-induced immunomodulatory efficacy. Reproduced with permission [96].

The pathogenesis of periodontitis is fundamentally directed by cell surface receptor-mediated signal transduction and intricate immune response mechanisms. Throughout disease progression, a multitude of signaling pathways, acting independently or synergistically, regulate macrophage polarization dynamics. Among these, the JAK/STAT pathway possesses a crucial impact on inflammatory regulation and macrophage activation. Specifically, the engagement of IL-4 and IL-13 with IL-4Rα receptors on macrophages activates the JAK/STAT6 signaling cascade, thereby driving macrophage polarization towards the M2 phenotype. Conversely, IFN-γ binding to its receptor on macrophages triggers STAT1 activation, promoting the production of pro-inflammatory cytokines and inducing macrophage polarization towards the M1 phenotype [91]. Recently, Qiao et al. reported that exosomes derived from dental pulp stem cells (DPSCs) could modulate macrophage phenotypes by inhibiting the IL-6/JAK2/STAT3 signaling axis [92]. As a consequence, this regulatory mechanism ameliorated the inflammatory microenvironment characteristic of periodontitis and promoted the regeneration of alveolar bone and periodontal epithelium in affected rats. Furthermore, given that the activation of Trem1 initiated the JAK2 signaling pathway, leading to STAT3 phosphorylation [93], Wu et al. first elucidated the critical role of the Trem1/STAT3/HIF-1α signaling pathway in orchestrating M1 polarization during periodontitis [94]. The genetic ablation of Trem1 in mice with periodontitis not only attenuated periodontal inflammation and alveolar bone loss but also decreased macrophage infiltration and reduced the M1/M2 macrophage ratio within lesion sites. Notably, the NF-κB signaling pathway is crucial for a variety of essential immune-related transcriptional programs [95], and its involvement in M1 polarization process has been confirmed [67]. As clarified by Tian et al., Nrf2 phosphorylation and subsequent NF-κB inhibition served as central mechanisms underlying the immunomodulatory function of baicalein-encapsulated mesoporous Prussian blue (MPB-BA) nanoparticles, which facilitated the switch of macrophages from M1 to M2 phenotype (Fig. 3A) [96]. In particular, immunofluorescence staining conducted in vitro detected a substantial reduction in the M1 macrophage marker CCR-7, alongside an enhancement of the M2 marker CD206, following MPB-BA treatment, irrespective of laser irradiation (Fig. 3B, C). According to histological validations in rat periodontitis models (Fig. 3D), MPB-BA intervention exhibited remarkable therapeutic efficacy by considerably suppressing inflammatory cell infiltration at both 3 and 7 days post-treatment (Fig. 3E). Moreover, micro-CT analysis further corroborated the significant inhibition of alveolar bone resorption (Fig. 3F). Mechanistically, immunohistochemical assessment (Fig. 3G) revealed that MPB-BA treatment led to an elevation of phosphorylated Nrf2 (p-Nrf2) while downregulating the expression of phosphorylated P65 (p-P65). Overall, these findings underscore that the modulation of the Nrf2/NF-κB axis is integral to the constructive role of MPB-BA, which in turn drives macrophage polarization towards an anti-inflammatory M2 phenotype, ultimately attenuating inflammation and preserving bone integrity. Additionally, Xia et al. verified that berberine was able to induce M2 macrophage polarization by inhibiting the NF-κB pathway, thereby exerting therapeutic benefits in periodontitis associated with type 2 diabetes [97]. Furthermore, the central role of NF-κB in macrophage-mediated inflammation is corroborated by findings in sepsis models, where the cholinergic agonist GTS-21 was shown to promote M2 macrophage polarization and mitigate atrial inflammation by specifically inhibiting the NF-κB P65/NLRP3/C-caspase 1 signaling axis, resulting in reduced IL-1β release [98]. As one of critical signaling pathways regulating macrophages, the PI3K/Akt pathway, centered on Akt, compromises numerous upstream and downstream effector molecules. According to Tian et al., Semaphorin 3A (Sema3A) could substantially induce the transformation of M1 to M2 macrophages by activating the PI3K/AKT/mTOR pathway [99]. In LPS-induced periodontitis models, immunohistochemistry revealed a notable increase in CD206^+^ cells and a remarkable decrease in iNOS^+^ cells in gingival and alveolar bone tissues, indicating that Sema3A has robust potential to suppress inflammatory cell infiltration. Furthermore, Wu et al. delineated, using a mouse periodontitis model, that the inhibition of Akt2 diminished M1 polarization levels and triggered M2 polarization by deactivating the JNK signaling pathway, thereby reducing the release of pro-inflammatory mediators and effectively alleviating bone resorption [100]. Regarding the complex interaction between Akt and NF-κB signalling [101], Wang et al. elaborated that in an inflammatory environment induced by periodontal pathogens, exosomes derived from periodontal ligament stem cells transported abundant miRNA-143-3p to macrophages and inhibited PI3K/AKT signaling by suppressing the expression of the target gene PI3Kγ, which subsequently activated NF-κB signaling and ultimately promoted M1 macrophage polarization [102]. As identified by HE staining in a mouse model, miR-143-3p administration posed an increased risk of damage to the periodontal ligament, immune cell infiltration, and alveolar bone loss.

Collectively, macrophage polarization research lays a solid foundation for restoring tissue homeostasis and establishing a healthy microbial ecology under periodontitis conditions. Understanding the roles of IL-10, TGF-β, and signaling pathways such as JAK and PI3K in macrophage polarization contributes to the precise control of macrophage phenotypes, providing targeted approaches to manage periodontal inflammation and promote tissue recovery and health.

### Pulpitis

Dental pulp, a loose connective tissue enveloped in hard dentin, contains a rich network of blood vessels, nerves, and highly specialized odontoblasts responsible for mineralization [103]. These components have an essential impact on maintaining the vitality and homeostasis of teeth. Multiple factors such as caries and trauma can disrupt the integrity of pulp-dentin complex, with bacterial infection being the primary culprit, potentially leading to pulpitis or pulp necrosis, which affects the physical and mental health of patients and reduces their quality of life [104]. When damage to the tooth structure results in pulp exposure, various immune cells collaborate to defend against foreign invaders. Innate immune cells in dental pulp comprise macrophages, neutrophils, dendritic cells, and lymphocytes [105]. Macrophages are predominantly located in the central pulp area and interact with pulp cells through specific molecular mechanisms [106]. Notably, macrophages adhere to the surface of pulp cells via molecules such as cell adhesion molecule 1 and laminin γ1 and regulate pulp cell proliferation and differentiation through the PDGFβ and JAG1 signaling pathways. In turn, pulp cells modulate the bactericidal activity of macrophages through galectin-9 and maintain macrophage activity via molecules such as tyrosine-protein kinase receptors, chemerin receptor 23, and TNF receptor superfamily member 1A [107]. In addition, macrophages can regulate the differentiation of DPSCs through molecules including EphA3, Notch3, and granule protein and receive signals from DPSCs via receptors such as activin A receptor type I, bone morphogenetic protein receptor type 1A, and type 1B, establishing a complex intercellular communication network [108,109].

Inflammatory responses play a critical protective role during pulp infection, drastically influencing its healing and regeneration [110]. During caries progression, bacteria and their metabolites penetrate the pulp and interact with extracellular pattern recognition receptors such as TLRs. Subsequently, this recognition triggers a cascade of complex signaling reactions, with NF-κB activation being pivotal. NF-κB activation further stimulates the production of a wide range of inflammatory cytokines, which are of importance in the acute inflammation phase, assisting to combat infection and promote pulp tissue repair [111]. However, chronic or excessive inflammation can lead to severe infections and host tissue damage. Hence, regulating inflammation has emerged as a prerequisite, ensuring it remains within suitable limits to prevent unwarranted host tissue damage and promote optimal healing. As demonstrated by Huang et al. in moderate caries, the demineralization process reaching the mid-third of dentin led to reversible pulpitis, which was dominated by M2 macrophages [112]. Conversely, when extensive caries penetrated the inner third of dentin, where M1 macrophages prevail, an irreversible pulpitis occurred. In accordance with this, Kadowaki et al. disclosed that M1 macrophages rapidly responded to pulp exposure, followed by a rise in M2 macrophages, which promoted reparative dentin accumulation at the exposure site [113]. These findings emphasized the indispensable roles of macrophage polarization in pulp-dentin complex repair and regeneration, paving new therapeutic avenues and pointing out new research directions for pulpitis.

In the regeneration of pulp-dentin complex, in addition to dentin formation, the regeneration of blood vessels and nerve networks is crucial for restoring pulp nutrition and sensory functions. It has been reported that M2 macrophages, positive for lymphatic vessel endothelial receptor 1, dramatically elevated mRNA expression of angiogenesis markers *MMPs* and promoted the migration and angiogenesis of HUVECs by secreting VEGF-A [114]. This suggests that M2 macrophages elicit positive effects on pulp tissue remodeling, particularly in revascularization. It is noteworthy that macrophages also showed positive roles in regulating neuroinflammation [115], with M2 macrophages closely related to Schwann cells [116]. Mokarram et al. discovered that in nerve injury sites, the phenotype, not the number, of macrophages is decisive for nerve regeneration, indicating a direct association between macrophage phenotype and nerve regeneration outcomes [117]. According to Gao et al., at the early phase of pulpitis, M1 macrophages mainly infiltrated the trigeminal ganglia [118]. After two weeks, M2 polarization dominated, reversing the M1/M2 ratio, and M2 macrophages exhibited remarkable anti-inflammatory competency. Consequently, this phenotypic switch from M1 to M2 contributed to nerve repair in pulpitis. In line with these findings, Gu et al. confirmed through a coronal pulpotomy model that macrophage transition from M1-dominated to M2-dominated during pulp regeneration is critical for creating a conducive environment for tissue regeneration [119].

**Fig. 4:**
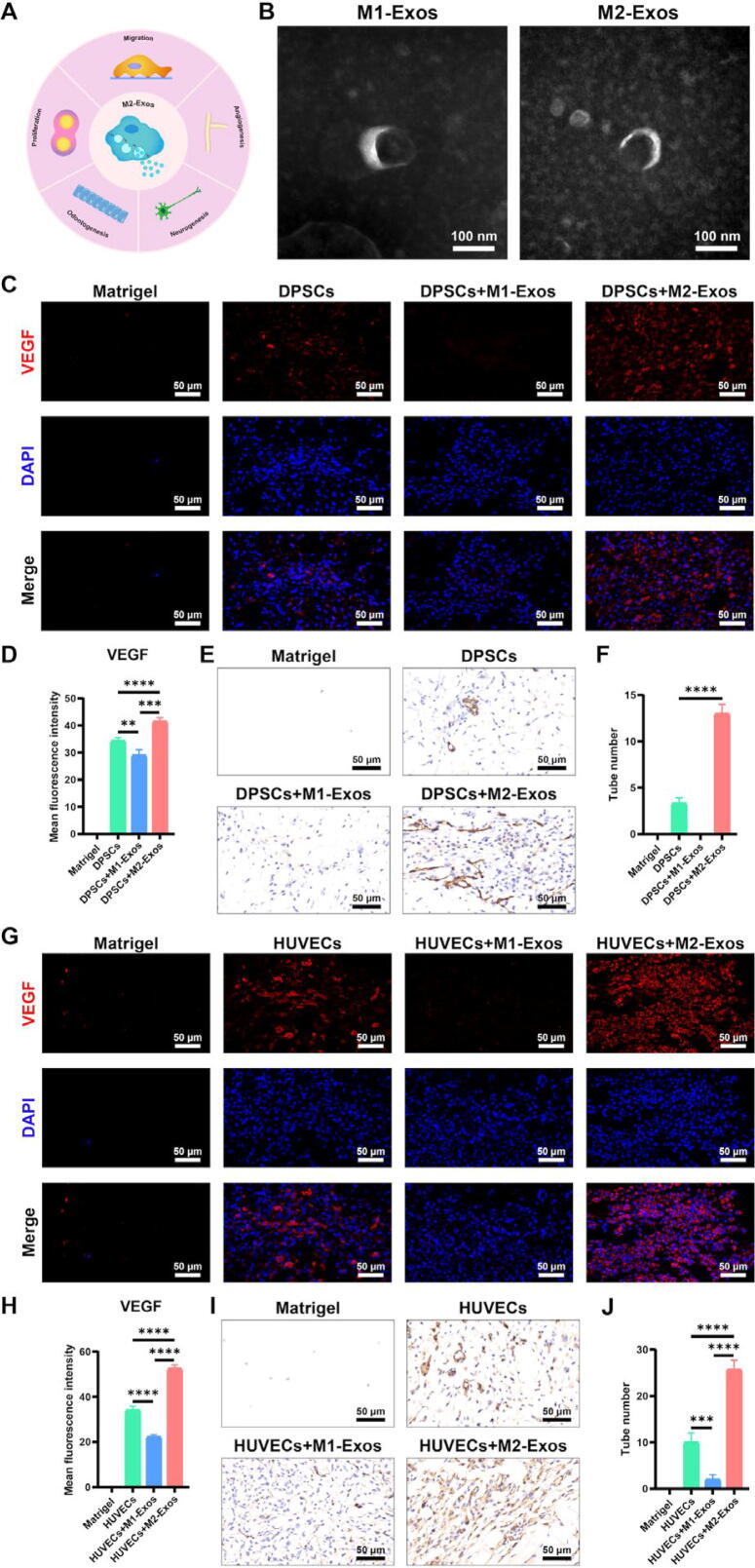
M2-Exos with superior pro-angiogenic potential facilitated vascular structure formation of DPSCs and HUVECs. (A) Schematic diagram showing the multifaceted roles of M2-Exos in orchestrating biological behaviors of DPSCs and HUVECs. (B) Both M1-Exos and M2-Exos possessed lipid-bilayer membranes, exhibiting saucer-like morphology with a diameter of around 100 nm. Scale bar: 100 nm. (C-J) In comparison with M1-Exos, M2-Exos exerted a promoting effect on the angiogenesis of DPSCs and HUVECs. (C, D) After transplantation with DPSCs encapsulated in Matrigel, M2-Exos substantially promoted blood vessel organization, as revealed by the remarkable expression of VEGF immunostaining compared with M1-Exos. (E, F) Consistently, immunohistochemistry staining for CD31 confirmed that M2-Exos elicited a notable enhancement in CD31-labeled tubular networks, indicating that M2-Exos hold immense potential to induce endothelial differentiation of DPSCs. (G, H) When imbedded in Matrigel with HUVECs, M2-Exos were capable of elevating the fluorescence intensity of VEGF, emphasizing the robust pro-angiogenic capability of M2-Exos. (I, J) In line with this, CD31-stained capillary-like lumens were considerably augmented in response to M2-Exo treatment. Scale bar: 50 μm. ^**^p < 0.01, ^***^p < 0.001, ^****^p < 0.0001. Reproduced with permission [121].

**Fig. 5:**
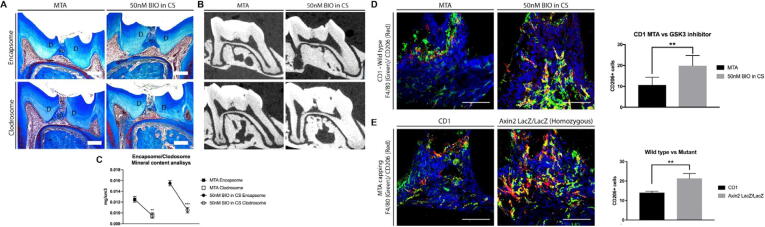
Wnt pathway activation facilitated the formation of denser reparative dentin by polarizing M2 macrophages. (A-C) Macrophage depletion posed a negative impairment in reparative dentin formation in mice. (A) As detected by Masson’s trichrome staining, mice subjected to Clodrosome injections and subsequent macrophage depletion displayed a significantly less dense reparative dentin formation in molars capped with mineral trioxide aggregate (MTA) or a GSK-3β antagonist, denoted as BIO, compared to the negative control group that received Encapsome injections. Scale bar: 100 μm. D, original dentin; RD, newly formed reparative dentin. (B) The results of the micro-CT analysis indicated a close correlation between macrophage depletion and limited accumulation of reparative dentin. (C) Quantification of mineral content provided additional evidence that depletion of macrophages markedly diminished the formation of reparative dentin. ^**^p = 0.0018, ^***^p = 0.0003. (D, E) Wnt pathway activation contributed to macrophage conversion from M1 to M2 phenotype. (D) In CD1 wild-type mice, anti-inflammatory M2 macrophages doubly stained by F4/80 and CD206 were notably increased in exposed molars capped with Wnt activator BIO, indicating that the activation of Wnt pathway promoted macrophage polarization during dental pulp repair and regeneration. Scale bar: 75 μm. ^**^p = 0.0033. (E) In comparison to wild-type mice, Axin2^−LacZ/LacZ^ mice, characterized by continuously elevated Wnt activity, demonstrated a remarkable enhancement in the transition of M2 macrophages. Reproduced with permission [125].

Focusing on the potential regulatory mechanisms of M2 macrophages in pulp-dentin complex regeneration, Zhou et al. illustrated that M2-CM considerably promoted DPSC proliferation, migration, and odontogenic differentiation in vitro and induced well-arranged collagen fibers in dentin-like tissue formation in vivo and enhanced the distribution of well-arranged collagen fibers in newly formed dentin-like tissue in vivo [120]*.* On the contrary, CM originating from M1 macrophages exerted a marked inhibitory effect on DPSC odontogenic differentiation, leading to disorganized collagen fibers. Consistently, Kadowaki et al. illuminated that M2-CM could induce DPSC differentiation into odontoblast-like cells, which thereby enhanced mineral deposition in a dose-dependent manner, suggesting that M2 regulate DPSC behaviors through soluble bioactive molecules or insoluble EVs [113]. In a recently published study, we revealed that M2 macrophage-secreted exosomes (M2-Exos) immensely ameliorated DPSC capability in odontogenesis, as indicated by promoted alkaline phosphatase activity, mineral nodule deposition, and the expression of odontogenic/osteogenic markers (Fig. 4A, B) [121]. Besides, M2-Exos remarkably facilitated neural-related gene expression and Nestin immunostaining intensity, highlighting their neuroprotective and neuroregenerative capacity. In terms of angiogenesis, subcutaneous transplantation experiments have demonstrated that DPSCs encapsulated in Matrigel and treated with M2-Exos facilitated more extensive vascular organization, as evidenced by markedly higher VEGF fluorescence intensity (Fig. 4C, D). Consistent with this, immunohistochemistry for CD31 showed a distinguishable enhancement in tubular network formation following M2-Exo intervention (Fig. 4E, F), indicating that M2-Exos effectively drove endothelial differentiation of DPSCs. Additionally, this pro-angiogenic effect was further corroborated in HUVECs, where the introduction of M2-Exos resulted in a substantial increase in VEGF expression (G, H) and a dramatic rise in CD31-stained capillary-like structures (Fig. 4I, J). Conversely, M1 macrophage-derived exosomes (M1-Exos) inhibited the aforementioned biological processes, shedding light on the crucial roles of M2-Exos in improving the pulp regeneration microenvironment with promising clinical prospects. In this regard, enormous efforts have been dedicated to inducing M2 polarization and propelling pulp regeneration. Of importance, the administration of macrophage mobilizing molecules, such as CGRP and CCL2, outstandingly promoted the polarization of macrophages towards M2 phenotypes, which encouragingly enhanced the proliferation of endothelial cells and pulp fibroblasts and increased pulp vascular permeability, thus facilitating pulpitis elimination and pulp regeneration [122]. As a classical signal transduction mechanism, the Wnt pathway is crucial in embryogenesis, cell differentiation, and stem cell generation [123]. Notably, the activation of Wnt signaling pathway could initiate the transition of macrophages from M1 to M2 [124]. According to Neves et al., the activation of the Wnt pathway using GSK-3β inhibitors gave rise to a significant reduction in apoptotic cells at the site of mouse dental pulp exposure, as suggested by TUNEL staining [125]. Intriguingly, a denser accumulation of reparative dentin (Fig. 5A–C) and noticeable elevation in M2 macrophages positively stained by F4/80 and CD206 in pulp tissue were also observed (Fig. 5D), emphasizing that Wnt activation-induced M1 to M2 polarization is beneficial for establishing an anti-inflammatory reparative environment in the early stage of pulp injury. Furthermore, compared to normal wild-type mice, the number of M2 macrophages increased significantly at the molar pulp injury site in *Axin2*^−LacZ/LacZ^ mice (Fig. 5E), which showed enhanced endogenous transduction of the Wnt pathway, confirming the positive influence of Wnt pathway activation on macrophage polarization in vivo*.* Therefore, stimulating macrophages to polarize more rapidly to the anti-inflammatory M2 state during pulp tissue regeneration might accelerate stem cell activation and repair processes, potentially serving as a therapeutic strategy for achieving functional healing and regeneration of pulp-dentin complex.

In conclusion, comprehensive study and accurate regulation of the inflammatory response, particularly macrophage polarization, will result in more efficacious strategies for treating pulpitis. This could improve the quality of life of patients and provide new approaches for pulp regeneration.

### Peri-implant inflammation

In the field of implant dentistry, the bone remodeling process after implant insertion is similar to tissue damage healing. However, the bone regeneration phase intends to be delayed to some degree due to the complex interaction between biological metabolism and tissue formation [86,126]. As foreign bodies, dental implants influence the bone immune microenvironment, thereby affecting anabolic and remodeling processes. Peri-implant diseases refer to infections and inflammations around dental implants, a comprehensive pathological state involving various inflammatory and infectious processes. Peri-implantitis is characterized by bone resorption around the implant, while peri-implant mucositis primarily encompasses inflammation of the soft tissues around the implant [127]. Peri-implant mucositis is often considered as a precursor to peri-implantitis [128]. A recent meta-analysis indicated that the prevalence of peri-implantitis in patients reached 19.53% [129]. Although the exact mechanisms underlying peri-implantitis remain elusive, mounting evidence has suggested that bacterial colonization around the implant and the resulting changes in the immune microenvironment stand as vital factors. Bacterial proliferation on the implant surface can polarize macrophages, triggering local inflammation and tissue damage around the implant [130]. In addition, macrophage polarization affects the repair and regeneration of local bone and vascular tissue, playing a critical role in the development of peri-implant disease [131,132].

**Fig. 6:**
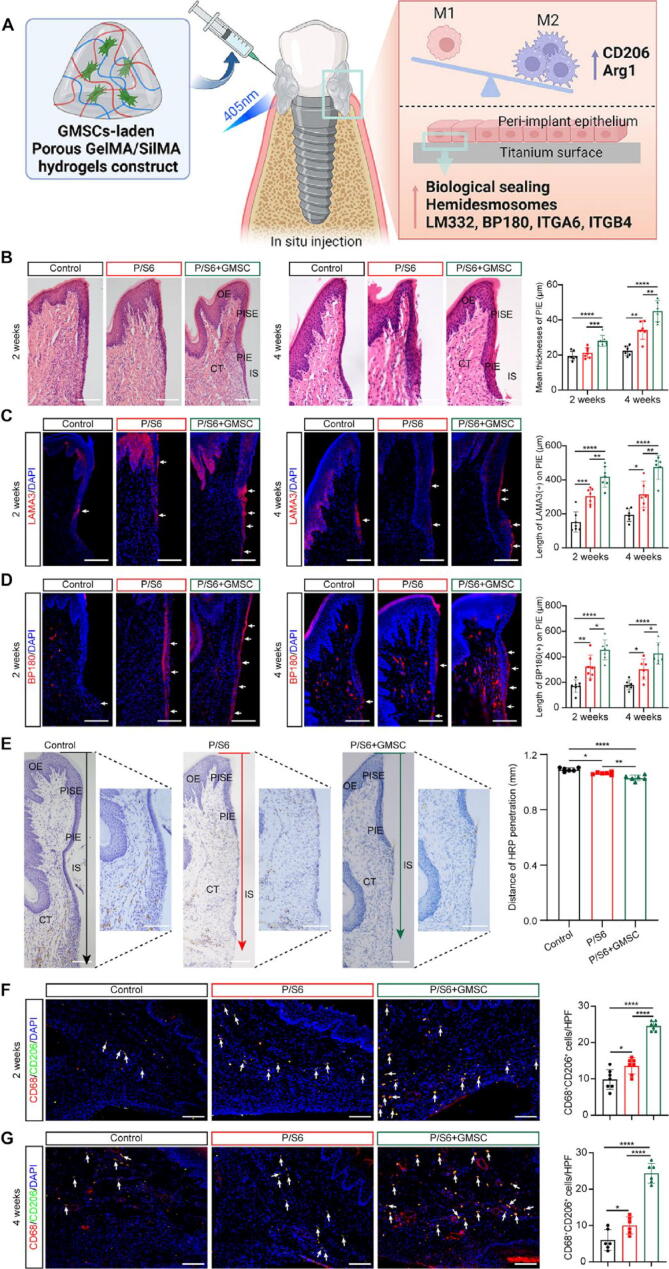
Porous GelMA/SilMA hydrogels encapsulating GMSCs enhanced per-implant epithelial sealing and drove M2 macrophage switch in rats. (A) Schematic illustration emphasizing the favorable effect of GMSCs-laden Porous GelMA/SilMA hydrogels on facilitating M2 macrophage polarization for promoted epithelial sealing. (B) As evidenced by HE staining, GMSC-laden GelMA/SilMA composite hydrogels, fabricated at the optimal proposition of 6: 1 (denoted as P/S6), significantly facilitated re-epithelialization around implants after 2- and 4-week treatment. (C, D) Immunofluorescence analysis demonstrated a more substantial and prolonged distribution of hemidesmosome characteristic proteins LAMA3 (C) and BP180 (D) at the implant-PIE interface in the GMSC-incorporated P/S6 hydrogel group compared to the other two groups after 2- and 4-week treatment, indicative of rapid epithelial reattachment. White arrows delineated the deposition of marker proteins along the implant-PIE interface. (E) When observed under a light microscope, GMSC-incorporated P/S6 hydrogels considerably diminished the penetration of HRP. (F, G) GMSC-incorporated P/S6 hydrogels exhibited a marked increase in the transition of M2 macrophages, as indicated by dual immunostaining of CD68 and CD206. White arrows pointed to doubly stained M2 macrophages. Scale bar: 100 μm. *p < 0.05, ^**^p < 0.01, ^***^p < 0.001, ^****^p < 0.0001. OE, oral epithelium; PISE, peri-implant sulcular epithelium; PIE, peri-implant epithelium; CT, connective tissue; IS, implant space. Reproduced with permission [137].

Previous studies have verified that peri-implantitis provoked substantial infiltration of inflammatory cells into the affected area, with an increase in M1 macrophages [130,133]. Especially in severe peri-implantitis, the amount of M1 macrophages was considerably elevated, which exhibited a marked positive correlation with greater probing depths [134]. It is also worth mentioning that macrophage types possess a remarkable effect on the healing mechanisms of peri-implant tissues in the short term after surgical treatment of peri-implantitis. As shown by immunohistochemical analysis, the polarization of the M1 phenotype and subsequent elevation of the M1/M2 ratio correlated with the severity of peri-implantitis [135]. Inspired by the structure of cecropin B, Zhang et al. designed a short antimicrobial peptide named KN-17 and validated that KN-17 was capable of guiding the polarization of macrophages towards M2 phenotypes via inhibiting NF-κB pathway and fostering inflammation resolution to reduce the occurrence of peri-implantitis [136]. Focused on the regulation of macrophage polarization and epithelial sealing around implants, Li et al. developed an injectable and photo-cross-linkable porous hydrogel network by combining gelatin methacryloyl (GelMA) and silk fibroin glycidyl methacrylate (SilMA) to incorporate gingival-derived MSCs (GMSCs) [137] (Fig. 6A). Encouragingly, this cell-laden polymer system significantly downregulated the gene and protein expression of pro-inflammatory-related M1 markers, including IL-6 and iNOS, while upregulating CD206 and Arg1 levels associated with anti-inflammatory M2 activation in vitro, thereby eliciting promising immunotherapeutic efficacy in inhibiting M1 polarization and enhancing M2 conversion. More importantly, when transplanted in rats around implants, GMSC-encapsulated GelMA/SilMA hydrogels remarkably accelerated re-epithelialization around implants at 2 and 4 weeks post-treatment, as shown by HE staining (Fig. 6B). Critically, immunofluorescence evaluation revealed a substantially enhanced and sustained deposition of hemidesmosome proteins, including LAMA3 (Fig. 6C) and BP180 (Fig. 6D), in epithelium surrounding implants, underscoring the rapid and robust reattachment of epithelial cells. Consistent with improved barrier function, this hydrogel system markedly attenuated the penetration of horseradish peroxidase (HRP) (Fig. 6E). In addition, dual immunostaining for CD68 and CD206 provided compelling evidence that this cell/hydrogel composite materials boosted a notable shift towards the anti-inflammatory M2 macrophage phenotype (Fig. 6F, G). Collectively, these findings suggest that the orchestrated actions of GMSC-laden GelMA/SilMA composite hydrogels facilitate the formation of mature and functional peri-implant epithelium, thereby strengthening the epithelial seal and reducing susceptibility to peri-implantitis. Hence, in order to prevent and promote the healing of peri-implantitis, the regulatory role of M2 macrophages should not be overlooked and deserves intense attention.

Furthermore, osteogenesis around the implant is crucial for its stability and function. This process primarily relies on the synergistic interaction of various cells, such as osteoblasts, osteoclasts, and macrophages [138]. Titanium particles released into surrounding tissues after implant placement can induce the recruitment of macrophages, which in turn shift to an M1 pro-inflammatory phenotype. This transformation triggers a massive secretion of inflammatory mediators, leading to local aseptic inflammation. As the inflammatory response continues and prolongs, marginal bone resorption around the implant is further promoted, ultimately resulting in unsuccessful osseointegration [139]. Notably, M1 macrophages can also directly regulate osteoclastogenesis by secreting inflammatory factors, especially TNF, to increase the number of osteoclast precursors and promote their differentiation into mature osteoclasts [140]. In sharp contrast, M2 macrophages produce favorable anti-inflammatory cytokines such as IL-10, suppressing early osteoclast development and promoting osteogenic differentiation of BMSCs [141]. Using rat tibia implant models, Zhao et al. proved that the subperiosteal injection of IL-1R-associated kinase 4 during the early inflammatory phase displayed a marked inhibitory role in osteoclast formation and activity by converting M1 to M2 macrophages [142]. Meanwhile, this process promoted osteogenic differentiation of BMSCs, significantly enhancing the bone-to-implant contact ratio and improving osseointegration. Increasing evidence has elaborated that the surface characteristics of implants, such as surface morphology and hydrophilicity, contribute to the regulation of macrophage behavior and immune responses [143]. Titanium is extensively employed in clinical practice due to its outstanding osseointegration capability. Different nanostructures of titanium surfaces elicit a regulatory impact on osteogenic differentiation of human osteoblasts and BMSCs in vitro [144]. As recently reported, modifying implant surfaces with titanium dioxide nanotubes displayed an enhancement in osseointegration [145,146]. Mechanistically, this beneficial effect could be achieved by regulating macrophage phenotypes around implants, which converted from M1 to M2. Graphene oxide (GO), an oxygen-derivative of graphene, has excellent mechanical strength, stable chemical properties, and good biocompatibility, and it has a wide range of applications in the biomedical field, especially in tissue regeneration. Using GO/IL-4 coatings, Li et al. achieved IL-4 delivery to macrophages from implant titanium substrates [147]*.* In vitro experiments revealed that the GO/IL-4 delivery system enormously promoted M2 macrophage polarization. In addition, conditioned medium from macrophages cocultured with GO/IL-4 system remarkably enhanced the proliferative, migratory, and osteogenic potential of BMSCs, as demonstrated by increased ALP activity and upregulated mRNA expression of *Runx-2*, *OCN*, and *osteopontin*. In vivo experiments confirmed that GO/IL-4 exerted a markedly promoting effect on inflammation resolution and osseointegration around the implant by modulating macrophage polarization, thus diminishing inflammatory cell infiltration, boosting new bone formation, and strengthening implant stability. Consequently, controlling macrophage polarization through cytokine addition or chemical coatings on implant surfaces can enhance implant-bone integration, improving implant stability and function.

In summary, conducting more in-depth research on macrophage polarization and its associated pathways and mechanisms offers novel insights and strategies to minimize the occurrence of peri-implantitis. Also, this is of vital importance for optimizing implant design and enhancing bone regeneration efficiency.

### Jawbone defects

Jawbone defects resulting from trauma, infection, tumors, or genetic diseases pose a significant challenge in clinical oral and maxillofacial surgery. These deformities not only result in changes to patients’ aesthetic appearance but also contribute to impairments in mastication, deglutition, and respiration, significantly affecting their overall quality of life and psychological well-being [148]. The repair and regeneration of the jawbone involve intricate coordination between osteoblasts and osteoclasts. Moreover, monocytes and macrophages play a central role in this process. Monocytes and bone macrophages situated in the canopy structures above osteoblasts at sites of bone remodeling directly participate in bone repair. Chang et al. illustrated using a macrophage-Fas-induced apoptosis mouse model that the depletion of macrophages considerably decreased the number of osteoblasts and negatively impaired their osteogenic capability [149]. Additionally, in a mouse tibia defect model, the reduction of macrophages has been observed to impede intramembranous bone repair and inhibit osteoblast development [150]. Together, these findings emphasize the indispensable role of macrophages in successful bone defect regeneration. Furthermore, prior research has indicated that coculturing with M1 macrophages markedly attenuated the expression of osteogenesis markers such as ALP, RUNX2, COL1, and OCN and mineralization competency in osteoblasts or MSCs [151,152]. Regarding the hidden mechanisms, it is presumed that pro-inflammatory factors, such as TNF-α, secreted by M1 macrophages, instigate apoptosis of osteoblasts and MSCs, consequently hindering their osteogenic differentiation [153,154]. However, when cocultured with M2 macrophages, there was a substantial enhancement in their osteogenic capability [155], which could be attributed to osteogenic factors such as TGF-β1 produced by M2 macrophages [156,157].

In addition to soluble factors, M2 have displayed strong potential in facilitating bone regeneration through the secretion of exosomes. Zhou et al. elucidated that M2-Exos substantially alleviated the formation of osteoclast-like cells and the mRNA expression of osteoclast markers in RANKL-stimulated Raw 264.7 cells [158]. Subsequent investigation revealed that M2-Exos mitigated RANKL-induced osteoclast differentiation by suppressing the expression of *CSF2* gene and inhibiting TNF-α production, thus offering a new therapeutic strategy and novel regulatory pathways for the potential clinical application of M2-Exos in bone disorders. Besides, according to Zhou et al., M2-Exos displayed a remarkable promotion on the osteogenic differentiation of BMSCs, as indicated by augmented ALP and alizarin red staining, along with marked elevation in ALP and RUNX2 gene and protein expression [159]. Furthermore, micro-CT detection in a murine calvarial defect model confirmed successful regeneration of calvarial bone upon M2-Exo employment. Histologically, HE staining demonstrated the formation of a well-organized bone structure resembling natural bone in the M2-Exo-treated group, while immunofluorescence staining exhibited a significant increase in ALP and RUNX2 expression intensity. It is also worth pointing out that the osteogenic capacity of M2-Exos could be further facilitated by preconditioning with hydrogen sulfide. These findings suggest that exosomes originating from normal macrophages as well as preconditioned macrophages serve vital functions in the regulation of bone regeneration. Consequently, in the context of bone repair and regeneration, macrophages and their phenotypes have been delineated to play indispensable roles via producing soluble factors and insoluble exosomes. As such, the regulation of macrophage polarization holds the potential to introduce novel strategies for the treatment of jawbone defects.

**Fig. 7:**
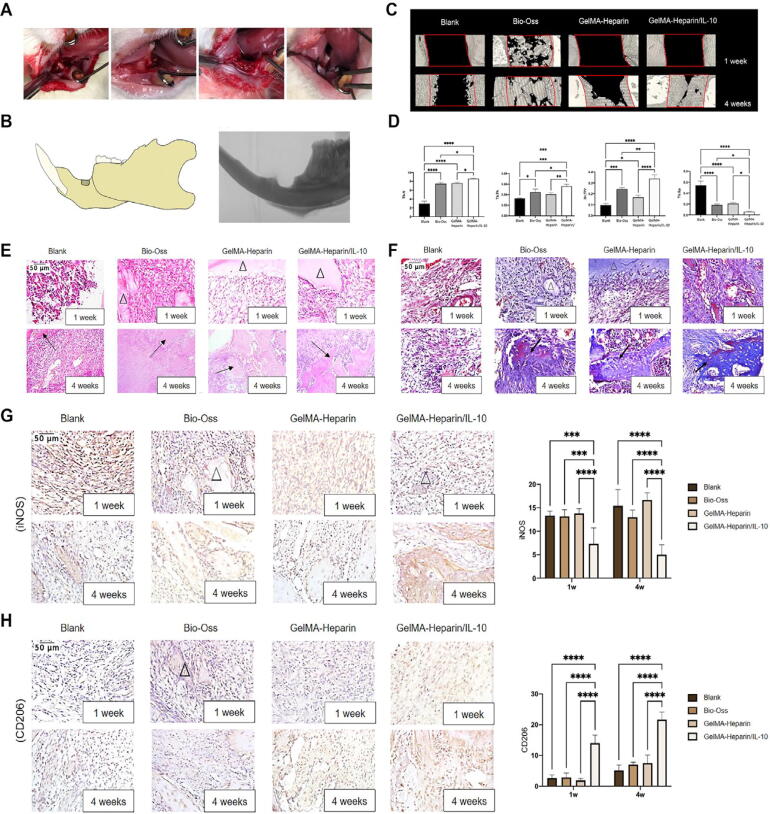
Controllable release of IL-10 from GelMA-heparin scaffolds facilitated the conversion of macrophages towards M2 phenotype. (A) Surgery process of jawbone defect establishment and composite scaffold transplantation. (B) Schematic diagram and micro-CT detection of bone defect model. (C) Micro-CT demonstrated that the combinatorial application of IL-10/GelMA-heparin exhibited the most substantial increase in new bone regeneration when compared to the other groups. (D) Quantitative analysis of newly formed bone structures ascertained the desirable promotion of IL-10-laden GelMA-heparin in mandibular defect regeneration. (E) As clarified by HE staining, IL-10-laden GelMA-heparin exerted a promoting effect on inflammatory cell eradication and osteoblast maturation. (F) Masson’s trichrome staining provided additional evidence that IL-10-incorporated GelMA-heparin scaffolds contributed to larger amount of blue-stained collagen and red-stained bone trabeculae. (G, H) IL-10/GelMA-heparin was superior to other counterparts in initiating the switch of macrophages from M1 phenotype to M1 fate. *p < 0.05, ^**^p < 0.01, ^***^p < 0.001, ^****^p < 0.0001. Δ indicated the remaining transplanted materials, and black arrows showed the newly formed bone trabeculae. Reproduced with permission [166].

Through a profound analysis of jawbone samples from patients with bisphosphonate-related osteonecrosis of the jaws (BRONJ), Wehrhan et al. discovered a significant elevation in macrophage density in BRONJ-affected tissues compared to healthy controls [160]. Notably, these macrophages predominantly exhibited an M1 phenotype, characterized by anti-regenerative and tissue-destructive functional properties. Further research pointed out that patients with BRONJ displayed compromised immunomodulatory capacity during wound healing and tissue regeneration, leading to a sustained predominance of M1-polarized macrophages at lesion sites with an apparent failure to transition to the reparative M2 phenotype. Accumulating evidence has suggested that therapeutic strategies aimed at promoting M2 polarization while inhibiting M1 polarization are beneficial to diminish disease severity and improve the treatment efficacy of BRONJ, which is expected to provide new ideas for therapeutic approaches [161,162]. Paschalidi et al. observed a dominance of the M2 phenotype in the early stages of medication-related osteonecrosis of the jaw (MRONJ) in patients without clinical infection, as illustrated by a higher density of M2 macrophages, a lower M1/M2 ratio, and significantly augmented IL-10 levels [163]. On the contrary, in patients with advanced MRONJ, macrophages showed a shift towards M1 phenotypes, reflected by increased M1 macrophage density, an elevated M1/M2 ratio, and marked overexpression of IL-6. Accordingly, designing and developing pharmacological agents or molecular interventions that modulate M2 macrophage polarization and thereby target the inflammatory microenvironment may constitute a novel strategy for treating jaw osteonecrosis. In this regard, Zhu et al. illuminated that zoledronic acid was able to induce nuclear translocation of NF-κB upon activation of TLR-4 signaling pathway in bone marrow-derived macrophages, thus promoting an upregulation of M1 macrophages in jawbone tissues around tooth extraction zone in mice [164]. Encouragingly, blockade of TLR-4 expression effectively abrogated NF-κB signaling, subsequently reducing M1 macrophage polarization and preventing BRONJ in mouse models. The critical role of the NF-κB pathway in sustaining a pro-inflammatory macrophage phenotype, as mentioned above, is further underscored by its involvement in other oral pathologies. In oral squamous cell carcinoma, the IKK2 inhibitor LY2409881 and the NEDD8-activating enzyme inhibitor MLN4924 have been demonstrated to effectively suppress tumor progression by inhibiting the NF-κB pathway. Given that NF-κB serves as a master regulator of M1 macrophage polarization, these agents may exert dual therapeutic effects by directly targeting cancer cells and concurrently modulating the tumor-associated macrophage compartment, potentially reprogramming them from a pro-tumorigenic M2 phenotype to an anti-tumorigenic M1 phenotype. This evidence reinforces the therapeutic rationale for targeting the NF-κB pathway to recalibrate macrophage polarization in inflammatory jawbone disorders such as BRONJ [165]. More recently, Hu et al. clarified that the application of IL-10 considerably induced the polarization of M2 macrophages, which consequently enhanced the migration and osteogenic differentiation potential of BMSCs in diabetic rats [166]. Following the establishment of jawbone defects in diabetic rat models (Fig. 7A, B), micro-CT analysis revealed that the sustained release of IL-10 from GelMA-heparin hydrogels led to the most pronounced enhancement of new bone formation compared to control groups (Fig. 7C, D). In histological terms, HE staining verified that the combination of IL-10/GelMA-heparin scaffolds remarkably facilitated the elimination of inflammatory cells and the maturation of osteoblasts (Fig. 7E). In line with this, Masson’s trichrome staining further highlighted a substantial increase in collagen deposition and structural integrity of the trabecular bone matrix (Fig. 7F). Of importance, the observed regenerative success was fundamentally attributable to the favorable immunomodulatory effects of IL-10/GelMA-heparin scaffolds, which exhibited a superior capacity to drive macrophage conversion from the pro-inflammatory M1 phenotype to the reparative M2 phenotype (Fig. 7G, H). Taken together, these findings indicate that IL-10 holds immense translational potential to enhance osteogenesis in jawbone defects by promoting M2 polarization, presenting innovative directions and strategies for managing jawbone defects in clinical scenarios.

Collectively, macrophages play a crucial role in immune-modulating osteogenesis due to their heterogeneity and plasticity, providing essential targets for the repair and regeneration of jawbone defects. M2 polarization, rather than M1 polarization, is more conducive to promoting the osteogenic process. However, given the complexity of macrophage functions, the specific cellular and biological mechanisms of M1 and M2 in jawbone regeneration require further exploration. Therefore, future research should focus on the particular mechanisms of macrophage polarization in jawbone regeneration in an attempt to provide new strategies for treating jawbone injuries.

## Insightful perspectives and conclusions

As a bacterial environment, the oral cavity encompasses a multitude of coordination and feedback mechanisms to maintain microenvironmental homeostasis, with macrophages serving a pivotal role. The polarization state of macrophages is indicative of their diverse and intricate functions. The precise and sequential polarization of M1 and M2 macrophages is imperative for promoting tissue repair and regeneration, which is essential for the treatment of oral diseases. M1 macrophages contribute to immune defense and injury responses, while M2 macrophages are involved in anti-inflammatory and reparative processes, thus constituting a crucial aspect of oral health. With advancements in research elucidating the regulatory mechanisms and signaling pathways of macrophage polarization, immunotherapy has shown promising results in the treatment of oral diseases. The key to alleviating inflammation and protecting oral tissues lies in balancing or sequentially controlling M1 and M2 macrophage polarization. This coordinated regulation enables the efficient elimination of pathogens, the prevention of ongoing inflammation, and the facilitation of tissue repair and regeneration. In this regard, there is an urgent need to conduct a comprehensive and detailed investigation into the mechanisms of action of various biomaterials, cytokines, and pharmacological mediators that govern macrophage polarization.

The behavior of macrophages can be selectively modulated by altering the chemical property, morphological structure, mechanical performance, and geometry of biomaterials, ultimately mediating the immune response. Certain metallic materials, such as titanium, gold, magnesium, and their alloys are commonly employed in oral and craniofacial surgeries for bone repair [167]. According to He et al., the nanoscale topology of implant surfaces could affect the polarization type of macrophages and exhibited great potential to trigger host inflammatory responses through particular signaling pathways distinct from conventional biochemical factors [168]. As illustrated by Choi et al., the surface modification of titanium implants by strontium-containing nanostructures considerably inhibited macrophage-mediated inflammatory response and elicited a remarkable promotion on osteoblast activity, thereby enhancing osseointegration [169]. In line with these findings, Hotchkiss et al. observed that smooth titanium surfaces induced M1 macrophage activation with augmented levels of IL-1β, IL-6, and TNF-α [143]. In sharp contrast, hydrophilic rough titanium surfaces promoted the activation of anti-inflammatory M2 macrophages, increasing levels of IL-4 and IL-10. In addition to metal materials, natural polymers, synthetic polymers, and their derivatives have been extensively studied for their capability to influence immune cell behavior during tissue regeneration. For instance, sulfated chitosan hydrogels containing type I collagen have been revealed to diminish M1 macrophage polarization in chronic diabetic wounds and promote the resolution of inflammation [170]. Furthermore, these hydrogels could suppress IL-6 production and facilitate the production of anti-inflammatory cytokines such as IL-4 and TGF-β1, which aided wound healing. Recently, Xuan et al. fabricated structurally ordered bredigite scaffolds using 3D technique and discovered that they were capable of promoting macrophage polarization towards M2 phenotypes, consequently enhancing the osteogenic differentiation and migration competency of BMSCs [171]. Notably, in rat cranial defect models, these 3D-printed bredigite materials markedly amplified M2 macrophage infiltration and the expression of osteogenic-related markers, which, therefore, fostered new bone formation.

Besides, a range of cytokines, growth factors, and chemokines can directly or indirectly promote macrophage infiltration and recruitment following tissue injury, thus regulating macrophage phenotypes and inducing M2 polarization [88]. It is worth noting that localized modulation of IL-4 concentration has been widely employed to stimulate the M2a phenotype, which in turn reduced inflammation and facilitated tissue regeneration [172]. Recently, Shehabeldin et al. utilized biodegradable polymeric carriers for the targeted delivery of IL-4 to inflamed periodontal tissues and elucidated through single-cell RNA sequencing that IL-4 effectively repressed pro-inflammatory pathways associated with tissue-destructive M1 macrophages [173]. Consequently, this intervention remarkably attenuated alveolar bone loss and boosted osteogenesis in murine periodontitis models, highlighting the therapeutic promise of IL-4 in the management of periodontal disease. As further elucidated by Ji et al., when encapsulated in thermosensitive hydroxypropyl chitin hydrogels functionalized with methacrylate groups, TGF-β1 has shown enormous potential in vitro and in vivo to shift recruited macrophages from the M1 to M2 phenotype [174]. This immunomodulatory strategy created a suitable microenvironment for MSC chondrogenic differentiation, achieving superior cartilage repair and regeneration. Furthermore, nucleic acid delivery approaches have emerged as promising modalities to finely regulate the balance between M1 and M2 macrophage polarization. In this regard, Wang et al. designed trisulfide-derived lipid nanoparticles to incorporate IL-4 mRNA [175]. Encouragingly, this formulation has demonstrated the capacity to reprogram M1 macrophages to anti-inflammatory M2 phenotypes in vitro by inducing IL-4 expression. As a result, administration of this system in a mouse diabetic wound model considerably accelerated wound healing by fostering the development of epidermal structures, angiogenic networks, and myofibroblast formation. In addition to mRNA, non-coding RNAs such as miRNA, siRNA, and shRNA have exerted crucial control over gene expression and cellular function in macrophages. For instance, miR-223 has been identified as a critical regulator in the NF-κB pathway, which governed macrophage polarization, indicating that the application of miR-223 could serve as an effective avenue for directing macrophage differentiation [176]. Aimed at circumventing the rapid degradation of siRNA with macrophages, Cui et al. have developed an innovative siRNA delivery platform, which compacted substantial quantities of siAkt2 stands into poly (L-lactic acid)-encapsulated nanoparticles, enabling robust and sustained intracellular siRNA release [177]. These nanoparticles were efficiently internalized by macrophages, facilitated lysosomal escape, and achieved continuous knockdown of Akt2 gene expression. This process drove metabolic reprogramming and considerably enhanced macrophage polarization towards the pro-regenerative M2 phenotype. Importantly, local administration of these nanoparticles in a periodontitis-induced bone defect model markedly induced M2 polarization and augmented alveolar bone regeneration, highlighting a promising therapeutic strategy for modulating macrophage function to promote tissue repair.

Exosomes, being efficient carriers of bioactive substances such as nucleic acids, proteins, and lipids, are also recognized as pivotal regulators of macrophage polarization under various pathological settings. There is growing evidence that exosomes derived from MSCs can effectively modulate macrophage phenotypic states [178,179]. Of importance, the capacity of exosomes in promoting M2 macrophage polarization can be strategically augmented by pretreating donor cells, loading pharmacological agents into exosomes, and employing genetic modification techniques. According to Wang et al., exosomes originating from TNF-α-preconditioned BMSCs could amplify M2 macrophage polarization while inhibiting M1 polarization, thereby considerably improving cardiac function, decreasing infarct size, and increasing left ventricular wall thickness in murine myocardial infarction models [180]. As indicated by Yan et al., exosomes secreted by icariin-loaded adipose stem cells elicited a remarkable suppressive potential on glycolysis through the ERK/HIF-1α/GLUT1 pathway, which facilitated the phenotypic shift of macrophages from M1 to M2 [181]. Furthermore, Liu et al. revealed that the administration of apoptotic EVs derived from PDLSCs into periodontal pockets of rats suffering from periodontitis substantially facilitated the transfer of mitochondrial components to macrophages via endocytosis [182]. The resulting mitochondrial functional reprogramming induced the repolarization of macrophages towards M2 phenotypes, which effectively modulated the local inflammatory microenvironment and restored macrophage homeostasis, ultimately contributing to periodontal tissue regeneration. More recently, Fu et al. have elaborated that M2-Exos engineered to express viral macrophage inflammatory protein II-lysosome-associated membrane protein 2b gene not only exhibited pronounced anti-inflammatory properties by modulating macrophage polarization, but also targeted the injured spinal cord, consequently diminishing the generation of pro-inflammatory factors and showing a promising neuroprotection role and functional recovery in mice [183]. Notably, macrophage-derived exosomes are intrinsically enriched with immunologically relevant molecules, including MHC proteins, cytokines, and non-coding RNAs, which precisely regulate gene expression and immune activity in recipient immune cells and facilitate antigen presentation [184]. Engineered macrophage-derived exosomes can further serve as versatile vehicles for delivering therapeutic payloads (e.g., drugs, siRNAs, proteins), thereby enabling the implementation of advanced combination therapies. Collectively, these investigations emphasize the immense competency of exosomes or EVs to regulate macrophage function and support immunotherapeutic intervention. Further research should prioritize the elucidation of underlying mechanisms and the translational application of exosome-based strategies for macrophage-targeted therapies.

Within the realm of macrophage-mediated disease regulation and immunotherapy, cutting-edge technologies are increasingly harnessing multifaceted approaches to precisely modulate macrophage phenotypes and the dynamic interplay of their microenvironments, thus exhibiting substantial translational potential for clinical applications in oral disease. Notably, the use of macrophage-derived vesicles has been extended to vaccine development. For instance, a bionic self-adjuvant vaccine platform constructed from pre-activated macrophage-derived vesicles demonstrated enhanced antigen delivery and retention, effectively synergizing innate and adaptive immune response and thereby establishing a novel immunomodulatory delivery paradigm [185]. In the field of gene editing, the CRISPR/CasRx (Cas13d) system, delivered via EVs as documented by Li et al., selectively targeted and inhibited the expression of pro-inflammatory genes in macrophages, significantly alleviating inflammation and organ damage in murine models of LPS-induced acute lung injury and sepsis [186]. These findings underscore the prospective utility of gene editing tools, such as CRISPR-Cas9, in modulating macrophage polarization through NF-κB knockout or IL-10 activation for the treatment of oral inflammatory diseases. Furthermore, optogenetics represents a promising avenue by integrating light-sensitive proteins, such as channelrhodopsin-2, with macrophage signaling networks to facilitate precise spatiotemporal control over cellular activities. This innovative approach holds the capability of inducing M2 polarization via targeted modulation of pertinent signaling pathways, which contribute to tissue repair within the oral cavity. Recently, accumulating studies have corroborated the efficacy of various photoactivators in downregulating the expression of pro-inflammatory cytokines derived from macrophages [[187], [188], [189]]. In addition, microbiome-engineered metabolic interventions possess remarkable competency to reshape immune microenvironments by recalibrating macrophage populations in oral tissues. Engineered probiotics, for instance, have been evidenced to suppress neutrophil infiltration, diminish levels of pro-inflammatory cytokines, and foster the polarization of macrophages towards anti-inflammatory M2 phenotypes [190,191]. Notably, in the domain of precision medicine, single-cell multi-omics technologies are instrumental in elucidating the transcriptomic and epigenomic signatures of macrophage subpopulations present in oral lesions. When integrated with artificial intelligence algorithms, these comprehensive datasets empower the development of patient-specific models for predicting therapeutic responses, thereby enabling the optimization of individualized combinatorial pharmacological regimens. Despite the substantial progress achieved with these innovative technologies, several challenges persist, particularly concerning the efficiency of therapeutic delivery within the complex oral environment, the safety profiles of engineered cellular entities, and the establishment of stringent standards for clinical translation. Beyond these technical considerations, the clinical implementation of macrophage-targeted therapies faces broader obstacles. These include the limited predictive capacity of preclinical animal models for human pathophysiology, the risk of systemic off-target effects when modulating ubiquitous widely distributed macrophages, the complexities of scalable manufacturing and quality control for advanced biological products (e.g., exosomes, engineered cells), and significant interpatient heterogeneity in immune responses. Addressing these multifaceted issues requires the development of smarter, tissue-targeted delivery systems, robust good manufacturing practice protocols, and biomarker-guided patient stratification strategies.

Taken together, emerging as a promising strategy for the treatment of oral inflammatory diseases, the combinatorial application of multiple strategies will contribute to establishing an appropriate immune microenvironment and modulating macrophage polarization. The concept of macrophage-mediated immunotherapy in oral tissue repair and regeneration has been extensively validated by numerous studies, thus opening up new avenues and directions for preventing and treating oral diseases. However, before widespread clinical translation and practice, it is essential to dedicate enormous efforts to illustrating the underlying molecular mechanisms as well as the long-term safety and soundness. In addition, more attention should be paid to understanding pathological characteristics and dynamics of macrophage polarization in the context of specific diseases. Last but not least, from the perspective of nomenclature, the M1/M2 dichotomy has served as a classical framework for macrophage classification in understanding macrophage activation. However, its inherent limitations have become increasingly evident in contemporary research. This dichotomous mode oversimplifies the continuum of macrophage activation states by only delineating the polarized extremes of pro-inflammatory (M1) and anti-inflammatory (M2) phenotypes, while simultaneously overlooking the existence of intermediate states, transitional phenotypes, and variations that are specific to distinct tissue environments. Furthermore, the archetypal definitions of these categories, which are predominantly derived from in vitro studies, insufficiently capture the complex spatiotemporal regulation exerted by the in vivo microenvironment on tissue-resident macrophages, such as influences from embryonic origin, duration of residency, metabolic reprogramming, and local inflammatory gradients. Recent investigations employing single-cell transcriptomics, multi-dimensional proteomics, and spatial metabolomics have illuminated the functional heterogeneity present within macrophage populations in various pathological contexts, such as atherosclerosis and malignancies, thus extending beyond the constraints of the M1/M2 paradigm [[192], [193], [194]]. In light of these insights, future classification strategies should adopt a more dynamic model guided by the “microenvironmental compass”, integrating cellular origin, tissue niche signals, temporal activation trajectories, and metabolic-epigenetic regulatory networks. Encouragingly, the incorporation of artificial intelligence-driven multi-omics data integration will further facilitate the construction of a comprehensive macrophage functional state map. This multi-dimensional classification system not only transcends the binary paradigm but also lays the theoretical groundwork for the advancement of precision-targeted therapeutic strategies. Such a sophisticated multi-dimensional classification system promises to transcend the binary paradigm, thereby providing a robust theoretical foundation for the advancement of precision-targeted therapeutic approaches. Overall, it is anticipated that macrophage modulation-based immunotherapies hold immense potential to become a reality in the treatment of oral inflammatory diseases.

## Declaration of competing interest

All authors declare no known competing financial interests or personal relationships that could have appeared to influence the work reported in this paper.
